# Cancer in pregnancy: FIGO Best practice advice and narrative review

**DOI:** 10.1002/ijgo.70258

**Published:** 2025-07-24

**Authors:** Surabhi Nanda, Melanie Nana, Long Nguyen‐Hoang, Sumaiya Adam, Fionnuala McAuliffe, Lina Bergman, Sarikapan Wilailak, Orla McNally, Cynthia V. Maxwell, Nikhil Purandare, Bo Jacobsson, Virna P. Medina, Anil Kapur, Titus Beyuo, Francisco Ruiloba, Ernesto Castelazo, Graeme N. Smith, Sharleen L. O'Reilly, Patrick O'Brien, Mark Hanson, Mary L. Rosser, Claudio Sosa, Valerie Guinto, Jonathan Berek, Catherine Nelson‐Piercy, Frédéric Amant, Liona Poon, the FIGO Committee on the Impact of Pregnancy on Long‐term Health, FIGO Committee on Women's Cancer, and the FIGO Division of Maternal and Newborn Health

**Affiliations:** ^1^ Women's Directorate, Guy's and St. Thomas' Hospital, Evelina London Children's Hospital King's College London London UK; ^2^ Faculty of Life Sciences and Medicine King's College London London UK; ^3^ Department of Obstetrics and Gynecology, Prince of Wales Hospital The Chinese University of Hong Kong Hong Kong SAR China; ^4^ Fetal Medicine Center Tam Anh HCMC General Hospital Ho Chi Minh City Vietnam; ^5^ Department of Obstetrics and Gynecology, School of Medicine, Faculty of Health Sciences University of Pretoria Pretoria South Africa; ^6^ Diabetes Research Centre, Faculty of Health Sciences University of Pretoria Pretoria South Africa; ^7^ UCD Perinatal Research Centre, School of Medicine, University College Dublin National Maternity Hospital Dublin Ireland; ^8^ Department of Obstetrics and Gynecology, Institute of Clinical Sciences, Sahlgrenska Academy University of Gothenburg Gothenburg Sweden; ^9^ Department of Obstetrics and Gynecology Stellenbosch University Cape Town South Africa; ^10^ Department of Women's and Children's Health Uppsala University Uppsala Sweden; ^11^ Faculty of Medicine Ramathibodi Hospital, Department of Obstetrics and Gynecology Mahidol University Bangkok Thailand; ^12^ Oncology and Dysplasia Services Royal Women's Hospital Melbourne Australia; ^13^ Maternal Fetal Medicine Sinai Health and Women's College Hospital, University of Toronto Ontario Canada; ^14^ University of Galway Galway Ireland; ^15^ Department of Obstetrics and Gynecology Sahlgrenska University Hospital, Ostra Gothenburg Sweden; ^16^ Department of Genetics and Bioinformatics, Domain of Health Data and Digitalization Institute of Public Health Oslo Norway; ^17^ Department of Obstetrics and Gynecology, Faculty of Health Universidad del Valle, Clínica Imbanaco Quirón Salud, Universidad, Libre Cali Colombia; ^18^ World Diabetes Foundation Bagsvaerd Denmark; ^19^ Department of Obstetrics and Gynecology University of Ghana Medical School Accra Ghana; ^20^ Fetal Medicine Research Institute King's College Hospital London UK; ^21^ International Federation of Gynecology and Obstetrics London UK; ^22^ Department of Obstetrics & Gynecology, Kingston Health Sciences Centre Queen's University Kingston Ontario Canada; ^23^ School of Agriculture and Food Science University College Dublin Dublin Ireland; ^24^ Institute for Women's Health University College London London UK; ^25^ Institute of Developmental Sciences University Hospital Southampton Southampton UK; ^26^ NIHR Southampton Biomedical Research Centre University of Southampton Southampton UK; ^27^ Department of Obstetrics and Gynecology Columbia University Irving Medical Center, New York‐Presbyterian New York New York USA; ^28^ Department of Obstetrics and Gynecology, School of Medicine University of Uruguay Montevideo Uruguay; ^29^ Department of Obstetrics and Gynecology, St. Luke's Medical Center Global City University of the Philippines‐Philippine General Hospital Manila Philippines; ^30^ Stanford Women's Cancer Center Laurie Kraus Lacob Professor Stanford University School of Medicine Stanford California USA; ^31^ Division Gynecologic Oncology UZ Leuven Leuven Belgium; ^32^ Department of Oncology KU Leuven Leuven Belgium

**Keywords:** abortion, cancer, chemotherapy, miscarriage, pregnancy, prematurity, radiotherapy, staging, termination

## Abstract

Cancer during pregnancy is relatively rare. The incidence is underestimated due to the lack of international registries covering both high‐income and low‐ and middle‐income countries, and is expected to rise with increasing maternal age and increasing global adoption of cell‐free DNA testing for aneuploidy. Physiological changes during pregnancy often make the diagnosis challenging and delayed. Lack of experience and knowledge about this condition may also contribute to late diagnosis, suboptimal management, and occasionally inadvertent fetal and/or maternal harm. The principles of cancer management in pregnancy for most cancer types do not differ significantly from the non‐pregnant population. The impact of investigations for diagnosis and staging, risks of surgery, systemic chemotherapy, and/or radiotherapy on fetal well‐being and preterm birth need to be considered for treatment and management planning, in addition to maternal wishes. Working in a multidisciplinary setting, ideally with medical and radiation oncologists, surgeons, radiologists, cancer specialist nurses, geneticists, psychologists, teratologists, and clinical pharmacologists, obstetricians, obstetric physicians, neonatologists, and experienced nursing and midwifery staff helps provide optimal care for the woman. This best practice advice aims to provide recommendations on the diagnosis and management of cancer in pregnancy, which can be adopted in all resource settings.

## 1 INTRODUCTION

**FIGURE 1:**
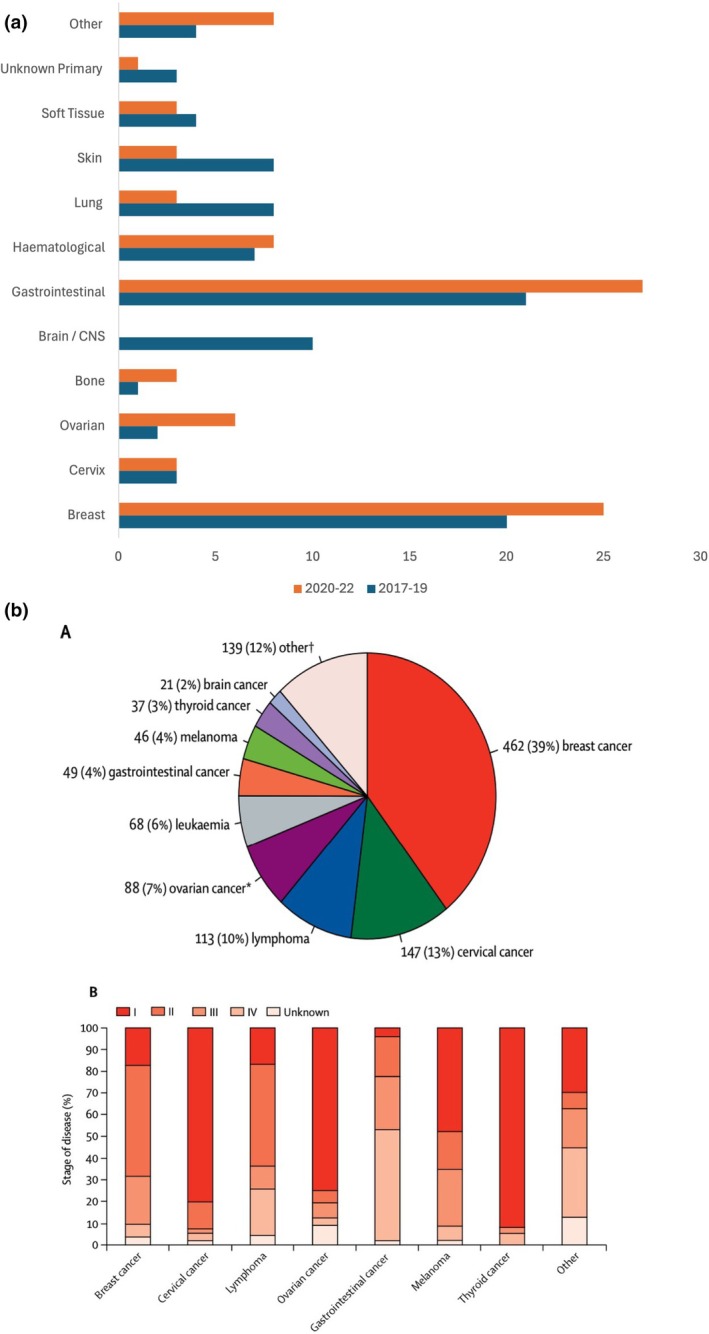
(a) Comparison of nature of the malignancies in the women who died in pregnancy to up to one year after delivery in UK and Ireland between 2017–19 and 2021–22.^9^, ^10^ (b) Data from the INCIP registry: (i) distribution of cancers during pregnancy and (ii) stage of disease at diagnosis by cancer type. Stage of disease was available for all solid cancers with TNM or FIGO classification. *Ovarian cancers include borderline ovarian tumors. ^†^Consists of 25 different cancer types.^13^ Reproduced from de Haan J, et al.^7^ with permission from Elsevier

Cancer remains the second most common cause of death for women of childbearing age.^1^ Although rare, the rate is predicted to increase with the older average childbearing age and with advances in non‐invasive prenatal testing for chromosomal abnormalities.^2^, ^3^, ^4^ In Europe, 2000–4000 pregnant women are diagnosed with cancer annually,^5^ with a global incidence between 17 per 100 000 live births and 25–27 per 100 000 pregnancies.^6^ It is, however, difficult to accurately establish the global incidence of cancer in pregnancy. This is due to disparity in reporting data in population‐based studies, a lower threshold for termination in these women due to lack of consensus in management, and a lack of health registries with matched oncology and obstetric data.^6^ The “International Network of Cancer, Infertility and Pregnancy” (INCIP; www.cancerinpregnancy.org) is a collaborative registry of 67 participating hospitals from 28 countries with standardized management and data collection protocols for women with cancer in pregnancy.^6^ Results from the INCIP database of 1170 women with cancer in pregnancy showed that breast cancer, hematologic cancers, melanoma, and cervical cancer are the most frequently diagnosed cancers during pregnancy, corresponding to the most common types of malignancy in women of reproductive age (Figure 1).^7^ Results from a UK‐based retrospective study of 119 women with cancer during pregnancy showed similar trends.^8^ Colorectal malignancies and lymphomas, compared to other forms of cancer, are usually detected in more advanced stages in pregnancy.^7^, ^9^, ^10^ Pregnancy‐associated cancer (PAC), which is defined as cancer diagnosed either during pregnancy or within 1 year of delivery, has been reported to have an incidence of 1 in 1000 pregnancies.^11^ A confidential enquiry into maternal death and morbidities in the UK reported a mortality rate of 0.87 per 100 000 pregnancies (95% confidence interval [CI] 0.52–1.35) in mothers with cancer in pregnancy, or within 6 weeks of giving birth,^9^ forming approximately 3% of all maternal deaths in the study period (2020–2022). Approximately 20% of these women entered their pregnancy with a history of past or recurrent cancer.^9^ Similarly, a recent analysis of 2359 women with cancer in pregnancy registered in the INCIP showed maternal mortality in 5.6% (131 women).^12^

**FIGURE 2:**
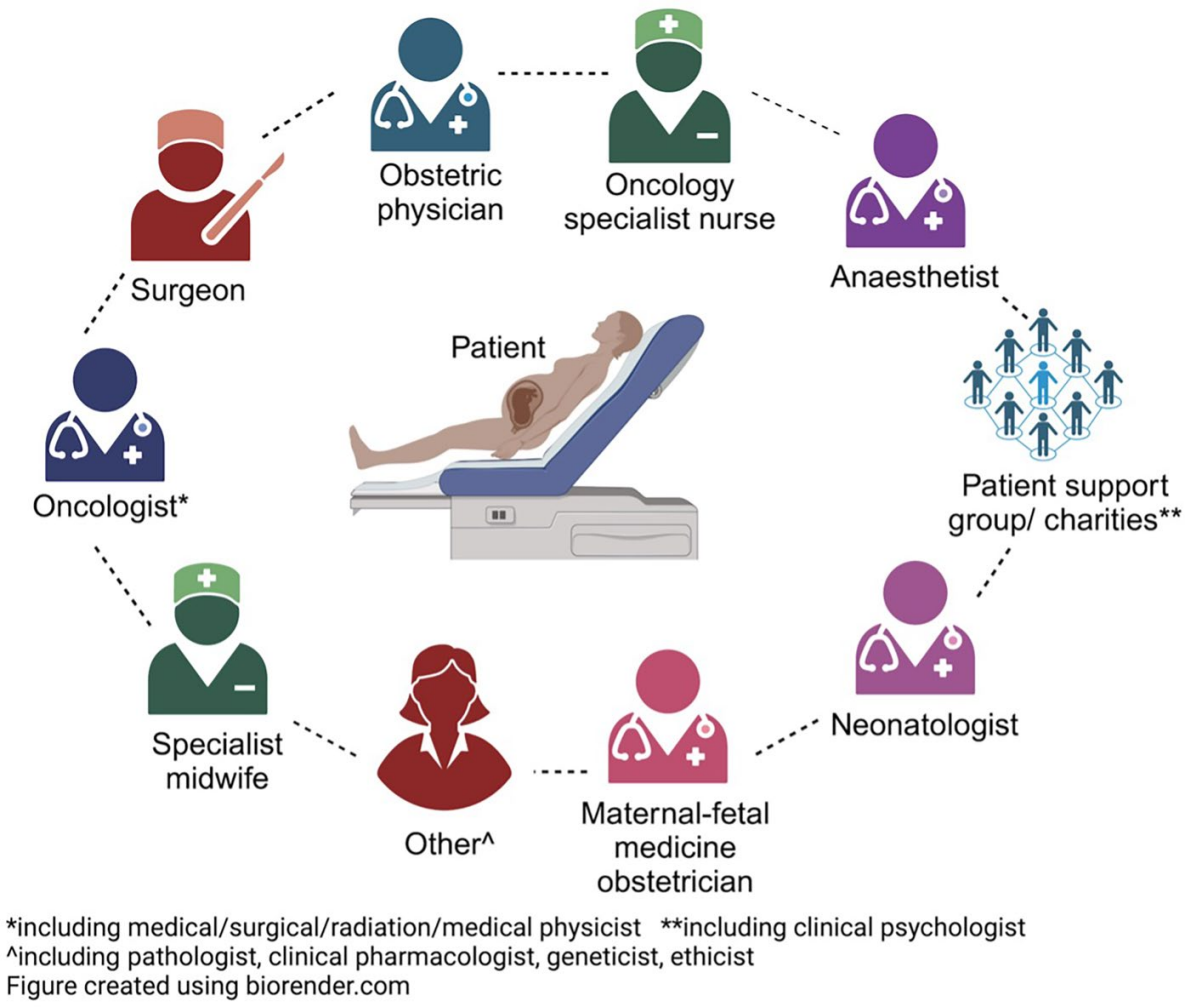
Multidisciplinary team input for women with cancer in pregnancy.

Pregnancies complicated by maternal cancer are high‐risk. Joined‐up woman‐centered care by a multidisciplinary team (MDT) comprising medical and radiation oncologists, surgeons, radiologists, cancer specialist nurses, geneticists, psychologists, teratologists, clinical pharmacologists, obstetricians, obstetric physicians, neonatologists, and experienced nursing and midwifery staff, as well as robust support for the woman and her family in the community, is the gold standard (Figure 2). It is acknowledged that not all MDT expertise is available across healthcare providers, thereby increasing the likelihood of inequitable care. The panel of assessors in the recent UK Confidential Enquiry concluded that in approximately 27% of women who died with malignancy, improvements to care may have made a difference in outcome.^9^ As cancer in pregnancy remains rare, most units would benefit from shared learning and peer support. The Advisory Board on Cancer, Infertility and Pregnancy (ABCIP; www‐ab‐cip.org) is a collaboration between different national advisory boards for clinical problems related to pregnant and non‐pregnant women with cancer who prefer to retain their fertility where possible and is easily accessible worldwide.^13^

The International Federation of Gynecology and Obstetrics (FIGO) is dedicated to the improvement of women's health and rights and to the reduction of disparities in health care available to women and newborns, in addition to advancing the science and practice of obstetrics and gynecology. The purpose of this best practice advice is to provide an evidence‐based approach for the management of women with cancer in pregnancy, in such a way that it can be adapted to various resource settings.

**Table:** 

Best practice advice: multidisciplinary team (MDT)
• Women with cancer in pregnancy should be managed by a MDT, with input from obstetricians. In addition, unbiased advice can be sought from international peer support groups
Pragmatic practice advice
Free MDT specialist resources like Advisory Board on Cancer, Infertility, and Pregnancy (ABCIP; www.ab‐cip.org) can be accessed for unbiased advice. The oncology plan should be conveyed to/discussed with the obstetric team, where joined‐up care is not practically possible

## 2 EFFECT OF PREGNANCY ON CANCER

The majority of data on the effect of pregnancy on cancer is mostly available for more common malignancies, such as breast cancer, in which pregnancy does not alter the course of cancer progression or impact on long‐term survival.^14^ Pregnancy can, however, cause delays in the identification of “red flags” of cancer. The symptoms of cancer may mimic physiological symptoms in pregnancy; therefore, some cancers may present at a later stage, thereby impacting the overall prognosis.^9^, ^10^, ^15^ Table 1 illustrates the common cancers seen in pregnancy and the effect of pregnancy on cancer management.

**TABLE 1:** Effect of pregnancy on commonly observed cancers in pregnancy.

Cancer	Effect of pregnancy on prognosis
Breast cancer (Figure 3)	Pregnancy does not worsen the prognosis for women diagnosed with breast cancer in pregnancy. Most pregnant women are diagnosed with infiltrating ductal adenocarcinomas, which are often associated with aggressive behavior (high incidence of grade 3 tumors, lymph vascular invasion, and estrogen receptor negativity) and may therefore be expected to have a poorer prognosis^18^ No increased rates of HER2 positivity^13^ Pathological features appear to be determined by age as opposed to a pregnancy effect^90^ There may be a delay in diagnosis with breast cancer in pregnancy, due to physiological changes of pregnancy‐engorgement, hypertrophy, nipple discharge, and increased density of breast tissue. This may lead to presentation with a more advanced disease at diagnosis^106^
Gastric cancer	Rare but pregnant women are at risk of delayed diagnosis as symptoms overlap with those of pregnancy Late diagnosis at more advanced stages thus carrying a worse prognosis and being overrepresented in terms of mortality^8^
Hodgkin lymphoma	No difference in 5‐year progression‐free survival regardless of disease stage, pregnant patients with Hodgkin lymphoma compared to non‐pregnant matched controls^26^
Cervical cancer	No conclusive evidence that pregnancy worsens the prognosis^29^ Insufficient evidence of impact of pregnancy on tumor biology^29^
Ovarian cancer	Insufficient evidence to suggest pregnancy negatively affects outcome^29^
Melanoma	No conclusive evidence that pregnancy worsens the prognosis^106^ Metastasis to the placenta and fetus is possible although the chance of this is very low^106^
CNS malignancies	Meningiomas and gliomas are the most commonly encountered brain tumors in pregnancy^107^, ^108^ In a systematic review of 316 patients with newly diagnosed (*n* = 202) and known (*n* = 114) gliomas in pregnancy, while pregnancy was found to provoke tumor growth on MRI, no effect of pregnancy on survival in low‐grade tumors was identified^94^

## 3 EFFECT OF CANCER ON PREGNANCY

### 3.1 Investigations and staging in pregnancy

#### Investigations

Delayed diagnosis can occur when there is reluctance to perform appropriate investigations due to concerns about maternal or fetal well‐being. Several factors have been implicated in such delays.^9^, ^10^ These include attributing abnormal symptoms—such as fatigue, nausea and vomiting, abdominal bloating, breast changes, vaginal or rectal bleeding—to normal physiological changes in pregnancy. Clinical inertia or reluctance to pursue investigations, including imaging studies with minimal radiation risk, also contributes.^9^, ^10^ Younger women are often perceived to be at lower risk for cancer, which may lead to malignancy being overlooked as a differential diagnosis. Occasionally, a woman's socioeconomic status, particularly where diagnostic procedures and treatments pose a financial burden, can preclude timely follow‐up.

The following best practice table on diagnosis provides guidance on the management of suspected cancer in pregnancy and includes recommendations from the recent Confidential Enquiries into Maternal Deaths in the UK.^9^

**Table:** 

Best practice advice: diagnosis
Pregnancy may delay presentation and diagnosis of some cancers due to overlap of symptoms with physiological changes Persistent symptoms necessitating recurrent presentations in pregnancy and increasing analgesia requirements should raise suspicion of malignancy and trigger investigations Women with symptoms suspicious of malignancy should be investigated, regardless of pregnancy status, in a timely manner as per locally agreed cancer pathways All women in early pregnancy should be asked about a previous history of cancer; if new symptoms present in pregnancy, medical advice and appropriate investigations should be organized All clinical staff caring for pregnant or postpartum women, whatever the location of care, are aware of the concerning “red flags,” and these should trigger early involvement of experienced/senior decision makers Symptoms of possible cancer or abnormal blood tests should prompt postnatal follow‐up Unexplained and recurrent vaginal bleeding irrespective of gestation should trigger a speculum examination and timely colposcopy referral, where indicated
Pragmatic practice advice
In low‐resource settings where all investigations are not available or there are concerns about safety to the fetus, an oncological MDT discussion should include obstetricians, the woman, and her partner for the most suitable investigations to be offered

Pregnant women presenting with concerning symptoms should not be denied appropriate investigations, including radiological imaging. Ultrasound and magnetic resonance imaging (MRI) are the preferred imaging modalities during pregnancy, as they do not involve exposure to ionizing radiation.^16^ Maternal risk is related to the theoretical increased susceptibility of breast tissue to ionizing radiation during pregnancy. However, a retrospective population‐based cohort study found no association between exposure to thoracic computed tomography (CT) during pregnancy or the postpartum period and an increased short‐term risk of maternal breast cancer (mean follow‐up duration: 5.9 years for the exposed group vs. 11.1 years for the non‐exposed group).^17^

If a cancer diagnosis is suspected, investigations should proceed in the same manner and on the same timescale as for a non‐pregnant woman. It would be reasonable to refer the woman for further investigation using a suspected cancer pathway referral (usually for an appointment within 2 weeks).^9^

The European Society for Medical Oncology (ESMO) guidelines recommend breast ultrasound and mammography for the primary diagnosis of breast cancer during pregnancy^18^; however, other imaging modalities would be recommended depending on the suspected cancer type and for staging. The main question before any investigation is how will the test results influence clinical decision‐making. A safe radiation dose (usually determined by a radiation physicist) in any diagnostic procedure does not increase the risk of stillbirth, congenital malformations, fetal growth restriction (FGR), or cognitive impairment.^19^ The risk to the fetus largely relates to an increased risk of childhood cancer (Table 2). In most cases where the fetal dose is 1 mGy or less, the associated risk of childhood cancer is less than 1 in 10 000, over and above the background risk of 1 in 500. Where the risk of fetal exposure is high, imaging should be undertaken only after a careful risk–benefit assessment, particularly when no suitable alternatives are available. When indicated, low‐dose CT or positron emission tomography (PET) scans may be considered in close consultation with a radiologist. It is essential to ask whether the result will influence clinical management.^20^, ^21^ Recent evidence suggests that shielding may increase fetal exposure and is therefore not recommended.^22^, ^23^, ^24^

**TABLE 2:** Maternal‐fetal risks and radiation doses with common imaging modalities.

Imaging modality	Maternal risks	Fetal risks		Contrast agents	Modifications required for lactation
		Typical fetal dose (mGy)	Risk of childhood cancer per examination (natural risk ~1 in 500)		
Ultrasound scan					
All	Nil	0	0	N/A	Nil
Ionizing radiation					
Chest radiograph	Theoretical increased susceptibility of breast tissue to effects of ionizing radiation in pregnancy and breastfeeding but this has not been confirmed in studies.^94^	0.001–0.01	<1 in 1 000 000	N/A	Nil
Lumbar spine radiograph		1.0–10.0	1 in 10 000 to 1 in 1000		Nil
Mammography		0.001–0.01	<1 in 1 000 000	Iodinated IV contrast agents cross the placenta but no evidence to suggest harm^95^	Nil
CT head		0.001–0.01	<1 in 1 000 000		Nil
CT pulmonary angiogram		0.01–0.1	1 in 1 000 000–100 000		Nil
CT thorax		0.01–0.1	1 in 1 000 000–100 000		Nil
CT abdomen		1–10	1 in 10 000 to 1 in 1000		Nil
CT abdomen and pelvis		10–50	1 in 1000 to 1 in 200		Nil
PET/CT whole body		10–50	1 in 1000 to 1 in 200		
Lung perfusion scan		0.1–1.0			
Magnetic resonance imaging					
All	Claustrophobia	0	0	Gadolinium should be avoided where possible, increased risk of rheumatological, inflammatory or infiltrative skin conditions, stillbirth or neonatal death^96^	

*Note*: Data summarized using the Royal College of Radiologists and the College of Radiographers “Protection of Pregnant Patients during Diagnostic Medical Exposures to Ionizing Radiation”.^19^, ^109^

Abbreviations: CT, computed tomography scan; N/A, not applicable; PET, positron emission tomography.

#### Staging

Pregnancy should not preclude staging investigations, which should be offered in a timely manner, in line with locally agreed cancer pathways for non‐pregnant women.^9^ Investigations such as chest CT or MRI should not be withheld due to pregnancy. For example, women with triple‐negative breast cancer and positive lymph nodes should undergo chest CT or MRI rather than chest radiography, as the latter lacks sufficient sensitivity to exclude lung or bone metastases.^9^ Regardless of gestational age, tissue and bone marrow biopsies should not be delayed.^14^, ^25^, ^26^ The histopathologist should be informed of the pregnancy and the suspected primary cancer, as physiological changes and gestational age may influence the interpretation of tumor pathology.^2^, ^27^, ^28^ Tissue biopsy (core needle or excisional) provides a more accurate diagnosis than fine‐needle aspiration cytology. Non‐pelvic lymph node biopsies can be performed throughout pregnancy, whereas pelvic lymph node dissection is limited to before 22 weeks of gestation.^29^ Sentinel lymph node mapping in breast cancer can be performed throughout pregnancy, ideally using a 1‐day protocol to minimize radiation exposure, which should remain below a cumulative dose of 5 mGy for the entire pregnancy. Technetium‐99 m colloid solution is preferred and can be administered 2 h preoperatively. Indocyanine green appears to be safe in pregnancy for fluorescence imaging, whereas blue dyes are discouraged due to the risk of anaphylaxis.^30^, ^31^, ^32^, ^33^, ^34^ Table 2 outlines fetal risk and radiation doses with common imaging modalities.

Tumor markers can be non‐specific, as they are outside of pregnancy, and their interpretation may be difficult due to physiological changes during pregnancy (Table 3).^35^

**TABLE 3:** Common tumor markers and changes in pregnancy.^35^, ^a^

Tumor markers	Associated histological types of ovarian and other tumors	Changes in pregnancy	Observations
CA125	Ovarian epithelial tumors	Increased in 1st trimester: (1) Starts at 30–40 days after LMP, peaks at 35–60 days (2) May reach 1250 U/mL (3) Decrease at the end of 1st trimester	Useful as tumor marker for ovarian epithelial tumors between 15 weeks of gestation and delivery
CEA	Epithelial tumors (particularly colorectal carcinoma)	Serum levels not influenced by pregnancy	Can be used as tumor marker in pregnancy
CA19.9	Gastrointestinal, pancreatic, and other adenocarcinomas; ovarian mucinous tumors	Mildly increased levels with increased gestational age, but never exceeding the normal range	Can be used as tumor marker in pregnancy
b‐HCG	Germ cell tumors (particularly choriocarcinoma)	Physiologically increased during pregnancy	Not possible to use as tumor marker during pregnancy
AFP	Germ cell tumors (endodermal sinus tumor, embryonal carcinoma, and mixed tumors)	Physiologically increased during pregnancy; abnormally increased in NTD and decreased in Down's syndrome	Serum levels usually <500 ng/mL in pregnancy complicated by NTD and >1000 ng/mL in germ cell tumors
LDH	Dysgerminomas	Increased in pregnancy, diseases (severe pre‐eclampsia, HELLP syndrome)	
Inhibin A	Granulosa cell tumors, mucinous carcinoma	Increased in 1st trimester (produced by developing placenta); abnormally increased in Down's syndrome	(1) Used in 2nd trimester for Down's syndrome screening (2) Increased levels require fetal and ovarian evaluation
He4	Serous, endometroid, and clear cell epithelial tumors	Lower levels in pregnant women; mildly increased in 3rd trimester compared to 2nd trimester; elevated levels can also be found in PTD	Promising tumor marker, but its value in pregnancy is not established

Abbreviations: AFP, alpha fetoprotein; LMP, last menstrual period; NTD, neural tube defect; PTD, preterm delivery.

^a^ Reproduced from open access publication, Cavaco‐Gomes J, Jorge Moreira C, Rocha A, Mota R, Paiva V, Costa A. Investigation and management of adnexal masses in pregnancy. Scientifica. 2016;2016(1):3012802.

**Table:** 

Best practice advice: staging
Pregnancy should not preclude full investigations, including biopsy, that would normally be performed outside of pregnancy for appropriate staging Gestational age should be considered when calculating radiation dose for investigations
Pragmatic practice advice
If a cancer diagnosis is suspected, investigations and staging should proceed in the same manner and on the same timescale as for non‐pregnant women

## 4 MANAGEMENT OF CANCER IN PREGNANCY: GENERAL PRINCIPLES

### 4.1 Surgery

Surgery can be performed when indicated, regardless of gestational age, although the early second trimester is preferable to reduce the risk of miscarriage.^36^ Surgery in the late second trimester or beyond may increase the risk of preterm delivery, placental abruption, and fetal distress, particularly during major abdominal and pelvic procedures.^2^ When open surgery is required later in pregnancy (beyond 20 weeks), compression of the inferior vena cava by the gravid uterus should be minimized by positioning the patient in a left‐lateral tilt (beyond 15 weeks for twin pregnancies). Surgical techniques for most cancers are similar in pregnant and non‐pregnant women. These include sentinel node biopsies and breast‐conserving surgery. Genital cancers are an exception. For cervical cancer, conization, simple trachelectomy, and pelvic lymph node or sentinel node resection are considered safe until the mid‐second trimester. Radical trachelectomy is not recommended during pregnancy. For advanced‐stage ovarian cancer, due to technical challenges posed by the gravid uterus, only a biopsy is typically performed during pregnancy, followed by neoadjuvant chemotherapy, with cytoreductive surgery postponed until after delivery.^2^, ^37^

Laparoscopic surgery should be preferred over open procedures when clinically indicated and where surgical expertise is available, as it is associated with fewer maternal and fetal complications compared to laparotomy. A limited intraoperative time (90–120 min) and low intra‐abdominal pressure (10–13 mmHg) are recommended.^29^ Direct trocar entry is usually preferred, as it reduces the risk of uterine perforation compared to standard umbilical entry using a Veress needle.^38^

The anesthetic approach needs to be adapted to account for the physiological changes during pregnancy and gestation, in addition to the usual considerations that should be taken into account when operating on a non‐pregnant cancer patient. Stable maternal blood pressure and oxygenation should be maintained as much as possible.^2^ Fetal heart tones should be auscultated before and after surgery, once the locally agreed gestational age for viability has been reached. Intraoperative cardiotocography (CTG) is usually not recommended. Similarly, steroids for fetal lung maturity and tocolytics may be considered if there is a high risk of preterm labor, especially when uterine manipulation is unavoidable. The neonatal team should also be informed. Adequate postoperative analgesia and hydration are vital.^29^ Thromboprophylaxis is recommended for at least 6 weeks postoperatively.^39^

### 4.2 Radiotherapy

Radiotherapy is usually avoided in pregnancy to reduce fetal risk. If needed, radiotherapy is possible with careful planning, after consulting with the pregnant woman, obstetric and oncology MDT, and in conjunction with the radiation specialists. The maternal and fetal consequences of treatment options with and without radiation, other treatment options, and gestational age should be discussed when planning radiotherapy during pregnancy. It is recommended that a physicist calculates a safe radiation dose, ensuring modification to the treatment plan (changing the field size, angle, and radiation energy). Pelvic radiation is not recommended in pregnancy. When non‐pelvic radiation is needed during pregnancy, appropriate shielding can ensure some fetal protection.^40^ Radiotherapy is typically limited to the first trimester, when the uterus remains distant from the irradiation field. A study of 68 maternal cases of radiotherapy during pregnancy across three centers found no neurocognitive, psychosocial, or chronic physical problems on follow‐up after birth.^41^

### 4.3 Chemotherapy

The ideal chemotherapeutic agent used in pregnancy should be effective while minimizing transplacental passage to ensure fetal safety. Physiological changes during pregnancy can affect drug distribution and pharmacokinetics, potentially influencing exposure and efficacy. Chemotherapy must be avoided during the first trimester (typically the first 12 weeks); after this period, most commonly used cytotoxic drugs are considered relatively safe.^42^ Standard chemotherapeutic agents used in non‐pregnancy regimes are recommended unless a specific agent is absolutely contraindicated during pregnancy (e.g. methotrexate, due to its association with miscarriage and increased risk of teratogenicity).^43^ Studies have shown comparable treatment outcomes for cancer (e.g. breast cancer) in pregnant and non‐pregnant populations receiving chemotherapy.^13^ Dosing is based on the mother's actual weight at the start of treatment, which is typically administered every 2–3 weeks depending on the agents or combinations used, and continued until approximately 34–35 weeks of gestation in preparation for delivery.^44^ This allows adequate time for drug washout and bone marrow recovery in both mother and infant between the final chemotherapy cycle and delivery, with plans to resume treatment postnatally if needed. This duration of the washout period depends on the specific agents or regimes used. Indwelling intravenous catheters for chemotherapy delivery carry a risk of thrombosis and infection; therefore, adequate nursing care and appropriate doses of prophylactic low molecular weight heparin (LMWH) are recommended while they remain in situ. Tables 4 and 5 summarize common chemotherapeutic agents and other management modalities used in the treatment of common cancers during pregnancy.

**TABLE 4:** Common chemotherapeutic agents used for treatment of common cancers in pregnancy.^45^, ^a^

Chemotherapy class and drugs	Placental transfer	Adverse events reported previously	Recommendations for clinical practice
Antimetabolites: cytarabine, fluorouracil, methotrexate	High risk (cytarabine^83^)	Congenital chromosome abnormalities with methotrexate and cytarabine^84^ Serious congenital deformities and death with cytarabine in the 1st trimester^85^ Multiple birth defects including congenital heart disease with methotrexate^86^	Cytarabine is the safest and can be used in the 2nd and 3rd trimesters to treat leukemia because risk to mother of delaying chemotherapy is high^85^, ^86^ Considered safe to use fluorouracil in the 2nd and 3rd trimesters^29^, ^87^ Always avoid methotrexate during pregnancy
Alkylating agents: busulfan, chlorambucil, cyclophosphamide, darcarbazine	Unclear	Cyclophosphamide embyopathy^b^ in the 1st trimester^87^, ^88^	Considered safe to use cyclophosphamide in the 2nd and 3rd trimesters^85^, ^89^ Considered safe to use dacarbazine in the 2nd and 3rd trimesters^2^, ^90^
Anthracycline antibiotics: daunorubicin, doxorubicin, epirubicin, idarubicin	Low risk (doxorubicin,^91^ epirubicin^92^) High risk (idarubicin,^93^ more lipophilc/smaller)	Transient dilated cardiomyopathy after maternal treatment with R‐CHOP^94^ Daunorubicin has the highest rate of adverse events (41%), including congenital malformations, acute respiratory distress, and myelosuppression with 1st‐trimester exposure^34^	Considered safe to use doxorubicin and epirubicin in the 2nd and 3rd trimesters; avoid idarubicin if possible^32^ Avoid daunorubicin use during pregnancy
Vinca alkaloids: vincristine, vinblastine, vinorelbine	No or low risk because of high protein binding nature^95^ (vincristine and vinblastine)	1st trimester: 2 separate cases of hydrocephalus and spontaneous abortion with vinblastine^96^	Single‐agent vinblastine has been noted to be relatively safe in the 1st trimester^95^, ^97^ as well as safe in 2nd and 3rd trimesters with other agent combinations^90^ Considered safe to use vincristine in the 2nd and 3rd trimesters^85^, ^89^ Limited data from case studies have shown vinorelbine to be safe to use in the 2nd and 3rd trimesters^98^, ^99^, ^100^, ^101^
Taxanes: paclitaxel, docetaxel	Not immediately, but delayed passage (paclitaxel,^83^, ^102^ docetaxel^102^)	Oligohydramnios with paclitaxel^103^	Considered safe to use taxane in the 2nd and 3rd trimesters^85^, ^104^; however, note that the NCCN does not recommend routine use in breast cancer given limited data^43^
Platinum agents: carboplatin, cisplatin, oxaliplatin	Low risk (cisplatin^105^, ^106^) High risk (carboplatin^83^, ^102^)	Ototoxicity with cisplatin^107^ Hypothyroidism with oxaliplatin^108^	Considered safe to use cisplatin in the 2nd and 3rd trimesters^85^, ^106^, ^109^ Considered safe to use carboplatin in the 2nd and 3rd trimesters^85^, ^104^ Considered safe to use intraperitoneal carboplatin in the 2nd and 3rd trimesters^110^ Limited data from case studies have shown oxaliplatin to be safe to use in the 2nd and 3rd trimesters^108^, ^111^, ^112^, ^113^

Abbreviations: NCCN, National Comprehensive Cancer Network; R‐CHOP, rituximab plus cyclophosphamide, doxorubicin, vincristine, and prednisone.

^a^ Reproduced from Silverstein J, Post AL, Chien AJ, et al. Multidisciplinary management of cancer during pregnancy. JCO Oncology Practice. 2020;16(9):545–557, with permission from Wolters Kluwer Inc.

^b^ Cyclophosphamide embryopathy phenotype: growth deficiency, developmental delay, craniosynostosis, blepharophimosis, flat nasal bridge, abnormal ears, and distal limb defects including hypoplastic thumbs and oligodactyly.

**TABLE 5:** Other modalities of management for cancer in pregnancy.

Modality	Examples	Considerations in pregnancy
Targeted agents^110^	Trastuzumab (Herceptin)^111^ (monoclonal antibodies)	Contraindicated in 1st trimester. With unintentional or accidental exposure, there is no indication for termination of pregnancy. Use. Any accidental use in pregnancy warrants very close monitoring for fetal well‐being and oligohydramnios. Ideally delayed until after delivery
	Rituximab^112^ (monoclonal antibodies)	Where indicated, e.g. R‐CHOP regime in non‐Hodgkin lymphoma, rituximab can be used from 1st trimester. There is safety profile for its use in other non‐malignant conditions in pregnancy
	Imatinib^110^ (tyrosine kinase inhibitors)	Used in management of Philadelphia chromosome‐ positive chronic myeloid leukemia. Reported teratogenicity in 1st trimester but appears to be safe during the 2nd and 3rd trimesters
	Bevacizumab^110^ (anti‐vascular endothelial growth factor and other antiangiogenic drugs)	High risk of teratogenicity and fetal loss; contraindicated in pregnancy
Hormonal therapy^113^	Tamoxifen (aromatase inhibitors)	Delay until after delivery
Immunotherapy^45^, ^114^	Ipilimumab (anti‐PD1/PD‐L1 agents)	Limited safety profile or long‐term follow‐up data; has been used in 1st trimester in some malignant melanoma cases

Abbreviation: R‐CHOP, rituximab plus cyclophosphamide, doxorubicin, vincristine, and prednisone.

### 4.4 Supportive and other treatments

Supportive medical treatment, with or without additional psychological support, is an integral part of cancer management during pregnancy.^45^ Antiemetics such as metoclopramide and serotonin receptor antagonists (e.g. ondansetron) are commonly used and considered safe during pregnancy. The safety of neurokinin‐1 receptor antagonists, such as aprepitant and fosaprepitant, has not been established. The use of betamethasone or dexamethasone as premedication is discouraged due to transplacental passage; instead, steroids with minimal placental transfer, such as methylprednisolone, prednisolone, or hydrocortisone, are recommended. Growth factors, including granulocyte colony‐stimulating factor and erythropoietin, are safe during pregnancy.^46^ Most analgesics, antacids, antihistamines, and anti‐microbial agents are considered safe and recommended as per non‐pregnancy drug choices, in consultation with the local pharmacy policies.^34^ Bone marrow transplant is contraindicated during pregnancy.^25^

**Table:** 

Best practice advice: management
Surgery can be performed whenever indicated and feasible, irrespective of gestational age Obstetric, anesthetic, and neonatal teams should be involved from the locally agreed gestational age of viability Radiotherapy, especially of the pelvis, is avoided during pregnancy where possible, but may be conducted in the first trimester Chemotherapy can be administered according to standard non‐pregnant regimens in most cases after the first trimester, with surveillance for fetal growth and preterm delivery, and monitoring of the mother for any toxicity or adverse effects Most supportive treatments while on chemotherapy are safe There are limited data on targeted therapy, although treatments can be individualized Biological agents can be used in pregnancy with MDT discretion The impact of delaying treatment, where needed, on maternal health, including mortality, should be clearly discussed and documented
Pragmatic practice advice
Maternal cancer treatment and continuation of pregnancy is to be preferred rather than medically induced (very) preterm delivery Where expertise is not available to deliver cancer care for a pregnant woman, an earlier transfer to a center that can support both maternal and fetal care is recommended. Failing this, a shared care model can be developed with support from a regional tertiary unit

### 4.5 Venous thromboembolism (VTE) prophylaxis

Active malignancy is associated with a significantly increased risk of VTE in pregnancy,^39^ with the odds of VTE being nearly seven times higher than those in pregnant women without malignancy.^39^, ^47^ Nearly 30% of women who died from cancer in pregnancy in the UK (2000–2022) experienced thrombosis or thromboembolism.^9^ All pregnant women with active cancer should be prescribed VTE prophylaxis using LMWH until 6 weeks postpartum, unless contraindicated.^39^ Previous studies have recommended initiating prophylaxis in the first trimester if additional risk factors are present.^48^, ^49^ Our recommendations regarding VTE are summarized in the best practice advice table below.

**Table:** 

Best practice advice: VTE prophylaxis
Risk assessment for VTE in early pregnancy is recommended for all pregnant women with cancer In any woman with metastatic disease, LMWH prophylaxis is recommended throughout the pregnancy and for at least 6 weeks postpartum Women with a diagnosis of active cancer should start LMWH prophylaxis from 28 weeks or from the first trimester if there are other risk factors, such as hospitalization, chemotherapy, nausea and vomiting in pregnancy, generally feeling unwell, immobility, or surgery Women with previous treated cancer do not need LMWH prophylaxis unless recurrence is diagnosed during pregnancy

### 4.6 Vaccine prophylaxis

Pregnant women with cancer, especially those undergoing treatment, are more susceptible to infection due to immunosuppression. All pregnant women should be offered influenza and SARS‐CoV‐2 vaccination, as well as tetanus vaccination where applicable.^50^, ^51^, ^52^ When planning cancer treatment, vaccines should ideally be administered before the initiation of systemic therapies. Live‐attenuated vaccines are contraindicated during pregnancy.^53^ Herpes zoster vaccination is recommended for patients receiver active cancer treatment. Screening for hepatitis B (HBV) and hepatitis C (HCV) should be undertaken in all pregnancies, and especially before starting anti‐cancer therapy. Patients with chronic HBV (HbsAg‐positive) undergoing treatment should receive antiviral prophylaxis during and for at least 12 months after the completion of treatment.^54^ Vaccinations such as whooping cough and respiratory syncytial virus (RSV), which primarily protect the infant, should not be withheld in women undergoing cancer treatment.^54^ Where indicated by local public health guidance, cancer or treatment for cancer should not be considered a contraindication for other vaccines, like hepatitis A and B, pneumococcal, meningococcal, yellow fever, Japanese encephalitis, polio, typhoid, and cholera.

## 5 OBSTETRIC CARE OF A PREGNANT WOMAN WITH CANCER

All pregnant women with current or recent cancer should be seen by an obstetrician in the first trimester.^9^, ^55^ Early pregnancy dating of pregnancy is vital, as it enables timely discussions about treatment options and decisions regarding continuation or termination of pregnancy.^9^, ^14^, ^29^ These decisions are influenced by individual circumstances, including psychological well‐being, cancer type, tumor biology, treatment stage, gestational age, and regional, legal, and ethical variations in obstetric practice. For example, there is limited experience managing advanced cervical cancer in early pregnancy, and termination may be appropriate. Aggressive hematologic malignancies in early pregnancy often require urgent treatment that may not be safe during pregnancy. Where available, consultation with a fetal medicine specialist is advised if accidental short‐term exposure to chemotherapy, biological, or supportive agents occur in the first trimester, although this does not always warrant a termination. Women should be informed that exposure to chemotherapy within the first 2 weeks after conception carries an increased risk of first‐trimester miscarriage.^56^ Current evidence does not suggest that termination improves maternal survival. However, it may be considered in cases of aggressive or advanced cancer diagnosed early in pregnancy.^12^ Reported termination rates in these cases (first and second trimester) range from just under 10% to approximately 25%.^8^, ^57^

Pregnancy in women with cancer—with or without treatment—is associated with increased risks of complications for both mother and newborn.^7^, ^56^, ^57^, ^58^ These cases warrant careful consideration and supportive multidisciplinary care under a named obstetric and oncology team^9^ (Figure 2). A recent meta‐analysis involving 44 262 cases of cancer diagnosed during pregnancy and 5722 within 1 year postpartum reported a two‐fold increase in the risk of pregnancy, fetal, and neonatal complications among these women.^59^

### 5.1 Screening for aneuploidies

Non‐invasive prenatal testing (NIPT) is increasingly being used as the most accurate screening method for fetal aneuploidies, including trisomies 21, 18, and 13.^60^ NIPT analyzes cell‐free DNA fragments in the maternal plasma, the majority of which originate from the mother, with a smaller proportion derived from fetal DNA.^61^ Consequently, NIPT can also detect genomic abnormalities in maternal DNA—including those associated with maternal cancers—which may confound the fetal risk assessment.^62^, ^63^, ^64^, ^65^

Cancers often exhibit somatic genetic alterations that are detectable in circulating cell‐free DNA.^66^, ^67^, ^68^, ^69^ Since many of these genetic derangements involve chromosomes 21, 18, and 13, it is possible that discordant NIPT results could be attributed to the presence of a maternal malignancy, especially when the NIPT results indicate multiple aneuploidies.^63^, ^64^, ^69^, ^70^, ^71^ Since the introduction of NIPT in prenatal screening, occult maternal malignancies have been reported as incidental findings after false‐positive NIPT tests.^71^ Various cancer types commonly encountered during pregnancy, such as breast cancer, lymphoma, and leukemia, as well as other cancers like ovarian cancer, multiple myeloma, digestive cancers, malignant melanoma, and sarcomas, have been accidentally identified through abnormal NIPT results.^62^, ^63^ In the general pregnant population, the rate of suspected occult malignancies detected through NIPT is in the range of 0.01%–0.02%.^72^, ^73^ However, when NIPT reveals multiple chromosomal aberrations, the likelihood of a maternal malignancy is approximately 20%–44%.^68^, ^71^, ^72^, ^73^ In such cases, a normal fetal karyotype and/or fetal sonogram may raise suspicion of a maternal cancer.^72^, ^74^ A referral for comprehensive oncologic examination is recommended, ideally guided by the specific tumor‐related indicators observed in the NIPT profile.^72^ Future innovative algorithms that consider the origin of cell‐free DNA, advanced approaches for measuring fetal fraction, and improved algorithms for aneuploidy detection may eventually enable the identification and exclusion of tumor‐derived cell‐free DNA and minimize the risk of misdiagnosis.^73^ Consequently, pregnant women with a known diagnosis of cancer should avoid undergoing NIPT as a screening tool for fetal aneuploidies.^74^ Instead, conventional combined first‐trimester screening with fetal nuchal translucency and serum biochemistry for common aneuploidies is recommended. In addition, detailed fetal anatomical ultrasonography should be considered.^74^

### 5.2 Low‐dose aspirin

Low‐dose aspirin is a safe intervention during pregnancy, effectively reducing the risk of pre‐eclampsia and other serious pregnancy complications.^75^, ^76^, ^77^ Although there is strong evidence for the use of low‐dose aspirin for the primary prevention several cancers, including colorectal cancer,^78^, ^79^, ^80^ there is limited evidence on the effect of aspirin on maternal cancer during pregnancy. It is also wort noting that administering low‐dose aspirin after a cancer diagnosis does not reduce cancer‐specific mortality or recurrence rates.^80^, ^81^, ^82^, ^83^ A meta‐analysis of 22 studies involving 1210 pregnant women with myeloproliferative neoplasms found that those who received aspirin and/or interferon therapy during pregnancy had nearly nine times greater odds of achieving a live birth compared to those who did not use aspirin (odds ratio [OR] 8.6, 95% CI 4.0–18.1; *I* ^2^ = 0%).^84^ We recommend that pregnant women diagnosed with cancer during pregnancy should follow current FIGO guidelines for the screening and prevention of pre‐eclampsia, including the use of low‐dose aspirin in those deemed high‐risk.^85^

### 5.3 Fetal surveillance

Maternal cancer during pregnancy is associated with an increased risk of preterm birth, FGR, stillbirth, and neonatal mortality.^86^ Therefore, close fetal surveillance is essential throughout gestation. In addition to the first‐trimester dating scan, a fetal anatomical survey is important, as the detection of fetal anomalies may influence the treatment plan.^14^, ^29^, ^86^, ^87^ Chemotherapy exposure during the first trimester has been shown to have a risk of fetal structural defects of up to 20%.^88^ However, a recent cohort study of 755 pregnant women with cancer found that major congenital malformations were significantly more likely when first chemotherapy was initiated before 12 weeks of gestation. In contrast, when chemotherapy was initiated after 12 weeks of gestation, the incidence of major congenital malformations was similar to that in the general population.^42^

Most chemotherapy agents cross the placenta and may affect fetal growth. Several large cohort studies have reported a high incidence of FGR, up to 21%, in pregnant women with cancer.^7^, ^59^ The duration of chemotherapy has been shown to have a negative impact on FGR.^116^ Consequently, it is recommended that fetal growth and amniotic fluid index are monitored through bi‐weekly ultrasound during antenatal chemotherapy.^14^, ^88^ Fetal Doppler assessment should be incorporated into fetal growth scans in cases of FGR or when evaluating for fetal anemia, particularly after exposure to platinum‐based chemotherapy agents. This includes measurement of the middle cerebral artery peak systolic velocity, which can help detect fetal anemia.^89^ Given the known association between chemotherapy, especially platinum and non‐platinum alkylating agents, and the risk of preterm contractions and delivery, we recommend regular monitoring of cervical length every 2–4 weeks in pregnant patients receiving antenatal chemotherapy.^7^, ^88^ In addition, for women who have undergone cervical conization, serial cervical length measurements are advised to monitor for cervical insufficiency.^90^, ^91^

### 5.4 Maternal surveillance

Maternal cancer is associated with an increased rate of pregnancy termination in both the first and second trimesters, as well as a higher rate of planned preterm birth, including early preterm birth before 32 weeks of gestation.^7^ Pregnant women with cervical cancer are more likely to deliver via cesarean section (CS), require blood transfusion, and undergo hysterectomy during their delivery admission.^91^ In addition to a higher risk of maternal VTE, women with cancer in pregnancy are also at increased risk of sepsis and severe maternal morbidity, as reported by Lee et al.^47^ A recent meta‐analysis of 22 studies showed a three‐fold increased risk of preterm birth among women with PAC, although there was no significant difference in the rates of preterm pre‐labor rupture of membranes (PPROM), gestational diabetes, or hypertensive disorders of pregnancy. That study also showed a 42‐fold increased risk of maternal death in those affected by PAC.^59^ Another meta‐analysis focusing on breast cancer in pregnancy reported that a 12‐week delay in treatment was associated with a 17% increase in maternal mortality.^92^ Early referral to a cancer specialist as per the non‐pregnant cancer pathway is therefore recommended.^9^

**FIGURE 3:**
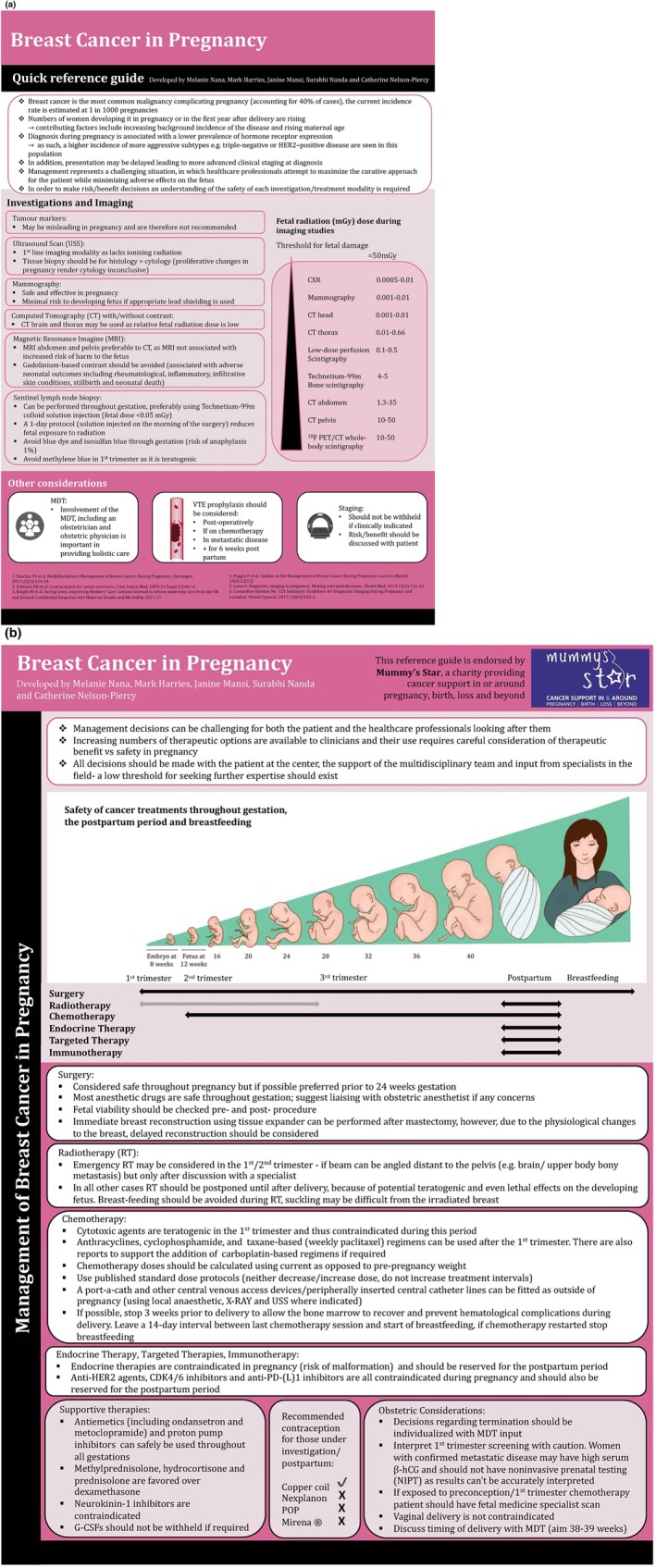
(a) Quick reference guideline for management of breast cancer in pregnancy. (b) Quick reference guideline for management of breast cancer in pregnancy.

Care of a pregnant woman with cancer is summarized in Tables 6A and 6B. An example of principles of managing breast cancer in pregnancy is shown in Figure 3.

**TABLE 6A:** Summary of management of cancer in pregnancy.

Management of cancer in pregancy: Synopsis of FIGO recommendations
*Diagnosis* Early pregnancy dating to ensure viability Obstetrician review in 1st trimester Referral to oncology teams as per non‐pregnant early cancer referral pathway (usually 2 weeks)
*MDT* Early discussion around options—continuation vs. termination of pregnancy If wider MDT is not feasible, clear plan to be communicated to obstetric teams ABCIP support where MDT teams or expertise is not available Respect wishes of the pregnant woman with cancer, including end‐of‐life care
*Staging and investigations* Offer as indicated as per non‐pregnant clinical indication (with some adjustements depending on gestational age) and within the same timeframe Inform pathologist of gestational age for review of any biopsies
*Treatment* Surgery Can be performed whenever indicated and feasible, irrespective of gestational age Left lateral tilt from around 20–22 weeks (earlier if multiple pregnancy) Obstetric anesthetic involvement Most surgical techniques (except genital cancer) similar to non‐pregnant cases Consider steroids and tocolytics around gestational age of viability if uterine manipulation or risk of preterm labor Chemotherapy From 12 weeks onwards every 2–3 weeks until 34–35 weeks of gestation Discuss association with SGA and preterm delivery Ensure 2–3 weeks of washout before delivery to allow call counts to recover Radiotherapy Can be used in 1st trimester for refractory cases with input from radiation specialists
*Pregnancy surveillance* Consider low‐dose aspirin if high risk in line with FIGO initiative on pre‐eclampsia Ultrasound Routine 1st trimester combined screening Do not recommend cell‐free DNA testing due to risk of false positives Fetal echocardiography on chemotherapy; consider fetal medicine review for anatomy scans if on cytotoxic treatment Fortnightly (2‐weekly) ultrasound for fetal well‐being while on treatment Cervical assessment (2–4 weekly) if risk of preterm labor Offer obstetric ultrasound to ensure fetal well‐being and rule out congenital malformations before treament Optimize general pregnancy health (e.g. nutrition, Hb, vitamin D, vaccines, infections, antiemetics, analgesics while on treatment). Maternal echocardiography if suspicion of chemotherapy induced cardiomyopathy
*Venous thromboembolism prophylaxis* VTE risk assessment in early pregnancy for all pregnant women with cancer If metastatic disease, LMWH for the whole pregnancy to at least 6 weeks postpartum If diagnosis of active cancer (primary or recurrence), LMWH from 28 weeks or from 1st trimester if other risk factors such as hospitalization, chemotherapy, nausea, and vomiting in pregnancy, generally unwell, immobility, or surgery Re‐evaluate VTE risk assesment in each trimester for other non‐malignancy related obstetric risk factors

Abbreviation: LMWH, low molecular weight heparin; MDT, multidisciplinary team; VTE, venous thromboembolism.

**TABLE 6B:** Summary of management of cancer in pregnancy.

Management of cancer in pregancy: synopsis of FIGO recommendations
*Delivery* Timing Avoid prematurity; plan for elective delivery >37 (usually 38+) weeks of gestation Washout of at least 3 weeks after the last 3‐week cycle of chemotherapy or 10 days after weekly chemotherapy Arrange delivery in a unit with suitable neonatal facilities if preterm Consider steroids for fetal lung maturity at least until 34 + 0 weeks of gestation Clear MDT plan around when to recommence/start treatment postnatally
Mode Offer planned vaginal delivery with induction of labor CS for obstetric indications or cancers like advanced cervical, vulval cancers, or intracranial tumors Offer early epidural with assisted second stage if raised intracranial pressure due to intracranial pathology Observe for and appropriately treat postpartum hemorrhage
*Postnatal care* Maternal Breastfeeding: encourage unless on chemotherapy. For non‐platinum chemoptherapy if formulat milk is not available, can breastfeed >3 days after dose Contraception: individualize options VTE prophylaxis Examine placental for histology to rule out potential metastasis Offer psychological support, including counseling, and support from independent charities, e.g. Mummy's Star Advice on deferring subsequent pregnancy (if applicable) Neonatal (within first 7 days of birth) Blood work: watch for neutropenia and abnormal biochemistry due to antenatal chemotherapy treatment Neonatal assessment for metastasis Pediatric (≥1 year of age) Genetic screening for cancer predisposition (if available) Monitor for cardiotoxicity (if anthracyclicine exposure in utero) Auditory assessment and follow‐up (if platinum derivative exposure in utero) Long‐term neurodevelopmental follow‐up

Abbreviation: MDT, multidisciplinary team; VTE, venous thromboembolism.

**Table:** 

Best practice advice
All women with cancer in pregnancy should be managed within a MDT with expertise in this area and should have a named obstetrician by the first trimester. Continuity of care within the MDT is recommended First‐trimester dating scan before commencing treatment, and second‐trimester anomaly scan and serial growth scans during treatment are recommended. A cervical assessment is recommended if the patient is at high risk of preterm labor All women on chemotherapy or other treatment should have vaccinations as per local policies NIPT testing should be avoided in women with known cancer in pregnancy Maternal risks of sepsis, thrombosis, preterm delivery, CS, and postpartum hemorrhage should be considered and discussed Fetal risks of miscarriage, structural defects if conceived on treatment, prematurity and related complications, low birth weight, and stillbirth should be considered and discussed Consider low‐dose aspirin if the patient is at high risk of pre‐eclampsia, in line with the FIGO initiative
Pragmatic practice advice
An antenatal care plan can be discussed with the regional unit or with advisory boards to deliver as much cohesive care as possible Usual antenatal care should not be compromised in women with cancer in pregnancy All women with cancer in pregnancy should be informed of their options for management from the first trimester, including the risk of preterm delivery and the option of termination of pregnancy

### 5.5 Perinatal care

Mothers with cancer in pregnancy have been shown to have increased risk of induction of labor (relative risk [RR] 1.36, 95% CI 1.10–1.67), postpartum hemorrhage (RR 1.30, 95% CI 1.06–1.60), and major puerperal infection (RR 1.79, 95% CI 1.10–2.91).^59^

### 5.6 Timing of delivery

Although chemotherapy is associated with a higher risk of preterm delivery, PPROM, and small for gestational age (SGA)/FGR neonates, whenever possible delivery should be planned for after 37 weeks (usually 38+ weeks) of gestation.^2^ This helps optimize fetal and neonatal outcomes, and facilitates the timing of postnatal treatment, where indicated. Unless contraindicated, vaginal birth is the preferred choice for the mode of delivery, although the CS rate in pregnant cancer patients is higher.^47^ Iatrogenic preterm delivery, which is common in this group,^47^ may be considered after multidisciplinary input (balancing risk of prematurity for the infant in favor of maternal outcome). This is especially the case in pregnant women needing end‐of‐life care, unstable or poor cancer control, and certain cancers like acute leukemia, intracranial tumors, or cervical cancer not responding to chemotherapy.^14^

Elective delivery should be planned so as to enable a suitable washout period of chemotherapy to avoid perinatal maternal and neonatal myelosuppression. Ideally, a washout period of 3 weeks is recommended for most 3‐weekly chemotherapy regimens. This washout period can be shorter (1–2 weeks) depending on fortnightly (bi‐weekly) or weekly regimens, as guided by the oncologists. Rates of transient neutropenia in neonates exposed to chemotherapy are in the range of 20%–30% within 4 weeks of chemotherapy to approximately 5% if delivered 4 weeks or more after stopping maternal chemotherapy.^45^ This is particularly important in preterm gestation, when neonates are more prone to both toxicity from chemotherapy agents as well as infections.^86^, ^93^

### 5.7 Mode of delivery

Vaginal delivery is generally recommended for most cancers in pregnancy; however, it is contraindicated in the majority of cases involving cervical, vulvar, rectal, and anal cancers. This is due to the risk of cancer cell implantation in the vaginal tear/episiotomy site, potential for obstructed labor, and the risk of dehiscence of malignant lesions postpartum.^29^ If indicated, simple or radical hysterectomy and pelvic lymphadenectomy may be performed concurrently at the time of the CS in these patients with prior discussion with the MDT.^43^

Delivery planning should also include discussions with obstetric anesthetists. Regional anesthesia is preferred, but where there is multi‐morbidity, women should be assessed for the suitability of a general anesthetic (GA). In women with intracranial tumors, early epidural is recommended. To minimize the risk of elevated intracranial pressure assocaied with intrapartum Valsalva maneuvers, CS under GA or an assisted vaginal delivery during the second stage may be considered.^94^ For women with bony metastasis, vaginal birth should be considered with careful planning with the MDT, due to the risk of long bone fractures.^2^

Delivery should be planned in a center that can offer care for the mother and that has neonatal facilities for a preterm infant. The timing of iatrogenic preterm delivery should consider the place of delivery and use of corticosteroids for fetal lung maturity, in line with FIGO guidance on steroids in preterm gestation.^95^ Due to the association of maternal cancer with SGA, especially with the risk of stillbirth and perinatal mortality in preterm SGA,^86^ we recommend continuous monitoring in labor. A large meta‐analysis of pregnancy‐associated cancers found that hematologic cancers were associated with the highest risk of intrauterine fetal death (RR 2.58, 95% CI 1.12–5.92), while breast cancer was linked to the highest risks of preterm birth (RR 5.62, 95% CI 3.53–8.94) and SGA infants (RR 5.92, 95% CI 4.41–7.95) compared with other cancers.^11^

Maternal health should be optimized before labor, including treating early signs of chorioamnionitis or subclinical infections. When women are at higher risk of pancytopenia (depending on the cancer or treatment), blood and platelet transfusions should be readily available to allow a safe vaginal delivery under regional anesthesia.^34^ Prophylactic thromboprophylaxis should be electively stopped at least 24 h before regional anesthesia. Certain cancers, especially those affecting the reproductive tract, increase the risk of hemorrhage during delivery.^7^

### 5.8 Placental examination

Placental pathology in women with cancer during pregnancy remains insufficiently studied. However, available evidence suggests a correlation between abnormal placental findings and SGA neonates, including placental vascular malformation and reduced placental size after maternal chemotherapy. Histologic examination of the placenta is essential and recommended to detect microscopic placental metastases and assess for potential fetal involvement, especially in cases of malignant melanoma or any metastatic malignancy.^14^

## 6 POSTNATAL CARE AND CONTRACEPTION

Postnatal care should be carefully optimized, considering both obstetric risk factors and those relating to the malignancy and its treatment. The timing of postpartum treatment initiation must be individualized to prioritize effective management of maternal cancer. Chemotherapy may commence from day 1 to up to 1–2 weeks after vaginal delivery and from day 8 to up to 3–4 weeks after CS.^45^ This short recovery period before starting treatment is vital for breastfeeding, bonding, and psychological well‐being. All women with a current or past cancer diagnosis should receive pre‐pregnancy counseling, including advice on contraception.^9^ Postnatal contraceptive planning should be individualized depending on the woman's wishes, circumstances, type of cancer, and proposed treatment (Table 7).

**TABLE 7:** Principles of postnatal discussion/pre‐pregnancy counseling for women with cancer.

Principles of postnatal discussion/pre‐pregnancy counseling for women with cancer
Women should be advised to postpone pregnancy for at least 2 years after a cancer diagnosis and completion of treatment as this is the period in which recurrence is most common^9^ Women and their partners should be counseled on appropriate contraception. For women with breast cancer only non‐hormonal contraception (barrier methods and the copper coil) are recommended in the UK. There are limited safety data on the use of levonorgestrel‐releasing intrauterine demise and women should be accordingly counseled^115^ Intrauterine copper device may be associated with a reduction in risk of cervical cancer^116^ If patients have received cancer treatments that could affect their fertility, they should be referred to a fertility specialist Women with inherited mutations linked to cancer such as BRCA1 should receive genetic counseling, including the possibility of pre‐implantation genetic testing

### 6.1 Breastfeeding

In addition to its health benefits,^96^ breastfeeding may help support the emotional and psychological well‐being of the mother with cancer in pregnancy. Women should be counseled that chemotherapy exposure can lead to a reduction in breast milk production due to lobular atrophy with fibrosis of breast tissue and breast infections.^97^ As such, breastfeeding during active chemotherapy treatment is not advised.^98^ This is primarily due to the risk of neutropenia in infants breastfed during cancer treatment, despite very low bio‐availability of chemotherapy in breast milk. It is recommended that there is a weaning period of at least 3 weeks between the last administration of most non‐platinum chemotherapy agents before initiation of breastfeeding, to mitigate the risk of neutropenia. The pros and cons of breastfeeding should be individualized, especially in a setting where formula milk is not available. There is limited information of the safety of chemotherapy metabolites in breast milk. However, it has been observed that the main chemotherapy molecules are hardly noticeable in breast milk after 48–72 h, apart from cisplatin, which remains detectable for a longer period. Therefore, where formula milk is unavailable, breastfeeding may be considered 3 days after chemotherapy administration. Breastfeeding is not recommended during treatment with non‐platinum derivatives and may be considered for a short period before commencing adjuvant endocrine therapy in hormone receptor‐positive breast cancer.^14^, ^34^ If there is a delay in starting chemotherapy during the weaning period, women can be encouraged to express and store breast milk. When breast feeding is contraindicated, lactation suppression with agents (e.g. cabergoline) is recommended. Some anti‐emetics used during chemotherapy may lead to breast engorgement. In women with breast cancer, reduced milk production from the affected breast is expected after breast‐conserving therapy; however, breastfeeding can be encouraged so long as the patient is not receiving chemotherapy drugs such as trastuzumab or tamoxifen.^14^, ^98^ Women using opioids for chronic pain should also be supported and encouraged to breastfeed.^99^

### 6.2 Neonatal and pediatric surveillance

There is no evidence that in utero exposure to chemotherapy results in significant long‐term health issues in offspring,^2^, ^42^, ^100^ including delays in neurological or psychological development.^100^, ^101^ Nevertheless, in utero chemotherapy exposure may still pose a theoretical risk of secondary malignancies necessitating the need for ongoing long‐term follow‐up of affected children.^97^, ^100^, ^102^ When maternal cancer is associated with a risk of placental or neonatal metastasis, neonatal teams should be informed in advance. Within the first 2–3 days of life, neonates should have a complete blood count and liver and renal function tests to rule out pancytopenia and other toxicities, especially those born less than 2–3 weeks after the last chemotherapy cycle. This is particularly important in preterm infants. Infants exposed to anthracycline in utero should undergo echocardiographic screening for cardiotoxicity within the first year of life, with continued monitoring every 3 years until adulthood. Children exposed to platinum‐based agents should receive auditory screening until the age of 5 years.^14^, ^29^, ^103^ Although there is no indication that secondary sexual characteristics are altered in children with in utero chemotherapy exposure, there is a theoretical risk of impaired fertility in children with exposed to gonadotoxic agents in utero.^41^, ^101^ Maternal death has been associated with adverse long‐term neurodevelopmental outcomes in affected children.^101^

## END‐OF‐LIFE CARE IN A PREGNANT/RECENTLY DELIVERED WOMAN WITH CANCER

Pregnancy or recent pregnancy with cancer should not be an exception for compassionate end‐of‐life care. It is essential that women are allowed to exercise control and autonomy over the end of their lives.^9^, ^12^ Women undergoing palliative care treatments should be allowed to spend time with their newborn. They may still be able to breastfeed, despite most pain‐relief regimes, which may provide additional psychological support.^9^, ^117^

## 8 PSYCHOSOCIAL IMPACT OF CANCER IN PREGNANCY

A diagnosis of cancer in pregnancy can be associated with significant psychosocial distress for both the pregnant woman and her family. While each mother's concerns will be different, a systematic review revealed that key risk factors for psychosocial distress include a mother's concerns about their infant's health and future and a woman feeling her condition is not well understood by healthcare professionals and support networks.^104^ The MDT should signpost the family to support groups and/or charities. Mummy's Star (https://www.mummysstar.org) is the only charity based in the UK and Ireland dedicated to women and birthing people diagnosed with cancer during pregnancy or within 12 months of giving birth but do provide international (non‐financial) support.^105^ They also provide support to families who, within the first year of birth, lose the partner who has given birth, as a result of cancer, and healthcare professionals who care for women, birthing people, and families facing this situation.

**Table:** 

Best practice advice
Women with cancer in pregnancy planning delivery should be counseled that there is a higher risk of CS and postpartum hemorrhage Delivery should be planned for 3 or more weeks after stopping 3‐weekly chemotherapy to enable recovery from maternal and fetal myelosuppression. Shorter interval (weekly) chemotherapy may need a 7–10‐day washout period Vaginal birth is recommended unless contraindicated Termination of pregnancy in the first or second trimester is a woman‐centered, MDT, and ethical decision and may be considered in advanced cancer, where treatment options safe in pregnancy are not the right choice for maternal benefit, upon maternal request, or where end‐of‐life care is being considered Continuous monitoring during labor is recommended, with a low threshold for initiating antibiotics at the first sign of infection The infant should be assessed by a neonatal team for infection, toxicity from chemotherapy, and for the presence of metastases where placental metastases have been identified End‐of‐life care should consider the mother and newborn's bonding time, which should not be withheld
Pragmatic practice advice
Ensure an appropriate washout period of chemotherapy before delivery Steroids for fetal lung maturity and delivery should be considered in a unit with neonatal support facilities when planning an elective preterm birth Breastfeeding is encouraged if the mother is not undergoing, or is awaiting the initiation of, postnatal treatment Charities like Mummy's Star can offer psychological support for mothers and families

## 9 CONCLUSION

Caring for pregnant women with cancer poses a complex medical, ethical, legal, and psychosocial challenge, highlighting the need for a multidisciplinary approach to optimize care. Table S1 summarizes four distinct clinical case studies, highlight the complex decision‐making process and the role of multidisciplinary boards in guiding treatment and management of cancer in pregnancy. Difficult decisions need to be made regarding appropriate investigation and treatment modalities and there may be inherent ethical dilemmas in balancing maternal and fetal well‐being, including whether to continue with the pregnancy. The complexity and rarity of cancer in pregnancy makes high‐quality data generation from randomized clinical trials almost impossible. There is global inequity in trained and interested MDT specialists to manage these women, and a lack of uniformity in available resources or guidance. This guidance provides an outline of management of this high‐risk cohort in pregnancy, and supports collaboration of national and international registries for maternal and pediatric outcomes for shared learning and advice (Tables 6 and 7). FIGO acknowledges the need for psychological support for these women and their families during and after pregnancy. It is important to empower the pregnant and recently pregnant women to have the same quality of care as any non‐pregnant person, to be properly informed, and to be involved in all decisions relating to their care.

## AUTHOR CONTRIBUTIONS

SN conceived the idea. SN, MN, CNP, LN‐H, LP, and FA contributed to the manuscript content and design. All authors contributed to manuscript writing.

## CONFLICT OF INTEREST STATEMENT

The authors have no conflicts of interest.

## Supporting information


Table S1.

## Data Availability

Data sharing is not applicable to this article as no new data were created or analyzed in this study.
