# Supplementary material for: Cancer in pregnancy: FIGO Best practice advice and narrative review
Source: Int J Gynaecol Obstet. 2025 Jul 24;171(1):131–51. doi: 10.1002/ijgo.70258 (PMC12447680; doi:10.1002/ijgo.70258)
Supplement: Supplementary file 1 — Table S1. [file IJGO-171-131-s001.docx]

**Clinical Vignettes**

| **Recurrent breast cancer in pregnancy**  **Case:** A 35-year-old, para 2, presented in her third pregnancy with a right-sided axillary lymph node. She had been diagnosed 2 years previously with high-grade DCIS of the right breast (5 × 6.5 cm). She had undergone mastectomy with close margins as well as postoperative radiotherapy. Her genetics were unremarkable. The new axillary lymph node was biopsied revealing a grade three triple-negative intraductal carcinoma. An MDT plan was to treat with six cycles of EC and LMWH prophylaxis in pregnancy; however, surveillance ultrasound revealed increased size of the mass after three cycles. An MDT was reconvened, and a decision was made to complete staging with an MRI of the liver and CT thorax and plan for surgery to remove the mass in pregnancy. This was done at 35 weeks of gestation after a fourth cycle of chemotherapy. After recovery from this surgery, she underwent an elective cesarean section at 38^+2^ weeks, for maternal request. Postoperatively, she commenced treatment with pembrolizumab, carboplatin, and paclitaxel before locoregional treatment.  **Reflection:** All pregnant women with a previous history of cancer should be asked about any new masses or symptoms. Chemotherapy can be used in pregnancy and surgery can be performed where indicated, which enabled this patient to carry her pregnancy to term. Appropriate staging investigations should not be omitted because of pregnancy to enable appropriate management plans. |
| --- |

| **Non-invasive prenatal testing for aneuploidy screening and presymptomatic detection of cancer**  **Case:** A routinely performed NIPT at 14 weeks in a 27-year-old asymptomatic primigravida showed an aberrant profile suggestive of maternal malignancy, confirmed by two independent samples. Clinical examination and blood sampling showed no abnormalities. Whole-body MRI documented a large diffusion restrictive mass in the anterior mediastinum and diffusion restrictive adenopathy in the left supraclavicular, lower jugular, and mid jugular regions. Lymph node excision biopsy confirmed the presence of classical nodular sclerosing type Hodgkin lymphoma, Lanugo stage II. At 20 weeks, 4-weekly chemotherapy administration, consisting of doxorubicin, bleomycin, vinblastine, and dacarbazine (ABVD) was initiated. Due to cervical shortening, cycle II day 15 was postponed. At 33 weeks, she developed cholestasis of pregnancy. After fetal lung maturation, labor was induced at 33^+5^ weeks due to elevated bile acids (>100 µmol/L). The patient gave birth to a healthy boy with a birth weight of 1935 g, who was admitted to the NICU for 23 days. PET-CT after delivery showed complete remission of the Hodgkin lymphoma and oncological treatment was completed after delivery with involved-field radiation therapy. After 2 years of follow-up, the patient remains in complete remission.  **Reflection:** NIPT allowed diagnosis and subsequent treatment of asymptomatic cancer during pregnancy. |
| --- |

| **Cervical cancer in pregnancy**  **Case:** A woman in her mid-30s, gravida 5 para 2, with a history of preterm pre-labor rupture of the membranes at 25 weeks in a previous twin pregnancy, presented with postcoital blood loss at 16 weeks in a spontaneous monochorionic diamniotic twin pregnancy. Examination showed a large ulcerative tumor on the anterior cervical lip and biopsy was performed. Pelvic MRI showed a tumor measuring 25 mm with no parametrial or pelvic wall invasion and no adenopathy. The patient was diagnosed with a FIGO IB2 invasive squamous cell carcinoma of the cervix. A laparotomic extraperitoneal pelvic lymphadenectomy was performed with 32 negative nodes. Neoadjuvant chemotherapy consisting of carboplatin and paclitaxel was administered at 18–30 weeks of gestation. After four cycles of chemotherapy, 6-weekly pelvic MRI showed stagnation of the oncological response. A classical cesarean section was performed at 34^+6^ weeks, and two healthy girls were admitted to the NICU due to preterm birth. The planned Wertheim hysterectomy could not be performed due to adhesions and after transposition of the right adnexa, the patient received curative pelvic radiotherapy with a total dose of 45 Gy and intracavitary brachytherapy.  **Reflection:** Neoadjuvant chemotherapy with close monitoring of the pregnancy and the oncological response of the cervical cancer during pregnancy is feasible and effective. |
| --- |

| **Bladder cancer in pregnancy**  **Case:** A 31-year-old woman in her first pregnancy was incidentally found to have a bladder mass at her routine 12-week dating ultrasound. A cystoscopy and biopsy at 15 weeks confirmed G3T1 transitional cell carcinoma with squamous differentiation. She was offered cystectomy, with possible hysterectomy in her local unit, without staging. A self-referral to tertiary oncology and obstetric unit led to a staging CT at 18 weeks, re-look cystoscopy and resection of the lesion. At 20–36 weeks, with multidisciplinary planning, she received four cycles of gemcitabine and cisplatin, with planned delivery at 36 weeks, after a 2-week washout period of chemotherapy. Delivery was a planned induction of labor. 8 weeks postnatally she had a cystectomy, and ileal conduit with vaginal/fertility-sparing and discharged home on day 8. She received continuity of care, with support from Mummy’s Star, and was able to breastfeed postnatally. She made an uneventful recovery, and is under oncology surveillance, with an ileal conduit, and no recurrence of cancer. Three years on, she has had pre-pregnancy counselling with obstetric medicine, maternal fetal medicine, and oncology specialist teams, as she is planning a second pregnancy soon.  **Reflection:** An incidental finding on ultrasound led to the diagnosis, and MDT input changed the outcome of the pregnancy and her oncology care. |
| --- |

Abbreviations: CT, computed tomography; DCIS, ductal carcinoma in situ; EC, epirubicin and cyclophosphamide; LMWH, low molecular weight heparin; MDT, multidisciplinary team; MRI, magnetic resonance imaging; NICU, neonatal intensive care unit.
